# Tandem repeats ubiquitously flank and contribute to translation initiation sites

**DOI:** 10.1186/s12863-022-01075-5

**Published:** 2022-07-27

**Authors:** Ali M. A. Maddi, Kaveh Kavousi, Masoud Arabfard, Hamid Ohadi, Mina Ohadi

**Affiliations:** 1 Laboratory of Complex Biological systems and Bioinformatics (CBB), Department of Bioinformatics, Institute of Biochemistry and Biophysics (IBB), University of Tehran, Tehran, Tehran 1417614411 Iran; 2 Chemical Injuries Research Center, Systems Biology and Poisonings Institute, Baqiyatallah University of Medical Sciences, Tehran, Tehran 1435916471 Iran; 3 School of Physics and Astronomy, University of St. Andrews, St. Andrews, KY16 9SS UK; 4 Iranian Research Center on Aging, University of Social Welfare and Rehabilitation Sciences, Tehran, Tehran 1985713871 Iran

**Keywords:** Genome-scale, Tandem repeat, Translation initiation site, Homology, TIS selection

## Abstract

**Background:**

While the evolutionary divergence of *cis*-regulatory sequences impacts translation initiation sites (TISs), the implication of tandem repeats (TRs) in TIS selection remains largely elusive. Here, we employed the TIS homology concept to study a possible link between TRs of all core lengths and repeats with TISs.

**Methods:**

Human, as reference sequence, and 83 other species were selected, and data was extracted on the entire protein-coding genes (*n* = 1,611,368) and transcripts (*n* = 2,730,515) annotated for those species from Ensembl 102. Following TIS identification, two different weighing vectors were employed to assign TIS homology, and the co-occurrence pattern of TISs with the upstream flanking TRs was studied in the selected species. The results were assessed in 10-fold cross-validation.

**Results:**

On average, every TIS was flanked by 1.19 TRs of various categories within its 120 bp upstream sequence, per species. We detected statistically significant enrichment of non-homologous human TISs co-occurring with human-specific TRs. On the contrary, homologous human TISs co-occurred significantly with non-human-specific TRs. 2991 human genes had at least one transcript, TIS of which was flanked by a human-specific TR. Text mining of a number of the identified genes, such as *CACNA1A, EIF5AL1, FOXK1, GABRB2, MYH2, SLC6A8,* and *TTN*, yielded predominant expression and functions in the human brain and/or skeletal muscle.

**Conclusion:**

We conclude that TRs ubiquitously flank and contribute to TIS selection at the trans-species level. Future functional analyses, such as a combination of genome editing strategies and in vitro protein synthesis may be employed to further investigate the impact of TRs on TIS selection.

**Supplementary Information:**

The online version contains supplementary material available at 10.1186/s12863-022-01075-5.

## Introduction

Translational regulation can be global or gene-specific, and most instances of translational regulation affect the rate-limiting initiation step [1, 2]. While mechanisms that result in the selection of translation initiation sites (TISs) are largely unknown, conservation of the alternative TIS positions and the associated open reading frames (ORFs) between human and mouse cells [3] implies physiological significance of alternative translation. A vast number of human protein-coding genes consist of alternative TISs, which are selected based on complex and yet not fully understood scanning mechanisms [3–6]. The alternative TISs can result in various protein structures and functions [7, 8].

While recent findings indicate that TISs are predominantly a result of molecular error [9], the probability of using a particular TIS differs among mRNA molecules, and can be dynamically regulated over time [10]. Selection of TISs and the level of translation and protein synthesis depend on the *cis* regulatory elements in the mRNA sequence and its secondary structure such as the formation of hair-pins, stem loops, and thermal stability [11–16]. In fact, the ribosomal machinery has the potential to scan and use several ORFs at a particular mRNA species [17].

A *tandem repeat* (TR) is a sequence of one or more DNA base pairs (bp) that is *repeated* on a DNA stretch. While TRs have profound biological effects in evolutionary, biological, and pathological terms [18–24], the effect of these intriguing elements on protein translation remains largely (if not totally) unknown. There are limited publications indicating that when located at the 5′ or 3′ untranslated region (UTR), short tandem repeats (STRs) (core units of 1–6 bp) can modulate translation, the effect of which has biological and pathological implications [25–29]. For example, eukaryotic initiation factors are clamped onto polypurine and polypyrimidine motifs in the 5′ UTRs of target RNAs, and influence translation [30]. Abnormal STR expansions impact TIS selection in a number of neurological disorders [31, 32].

Based on a TIS homology approach, we previously reported a link between STRs and TIS selection [33]. Here, we extend our study to TRs of all core lengths and repeats, an additional weighing vector (vector *W*_2_), several additional species, improved sequence retrieval methods, and a newly developed software and database for data collection and storage.

## Results and discussion

### TRs are ubiquitous *cis* elements flanking TISs

**Fig. 1:**
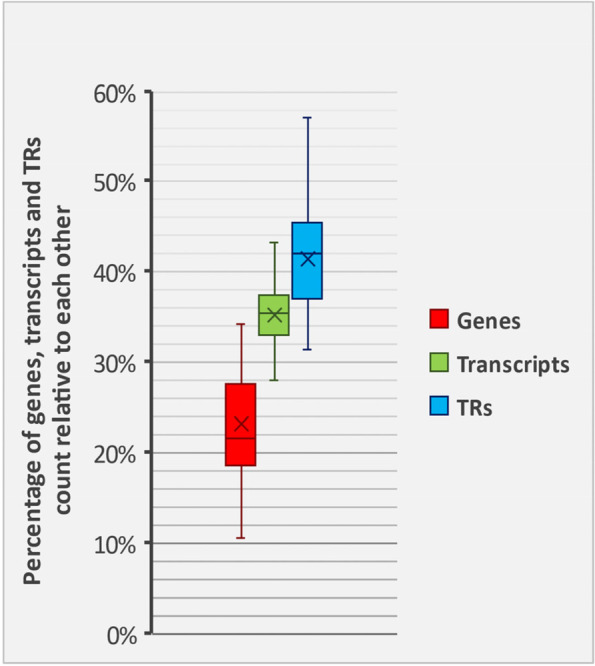
Abundance interval of the genes, transcripts, and TRs to each other. In this chart, we compared variations in the number of genes, transcripts, and TRs in different species relative to each other. The vertical axis shows what percentage of the total number of genes plus transcripts plus TRs in different species belong to the genes, transcripts, or TRs

**Fig. 2:**
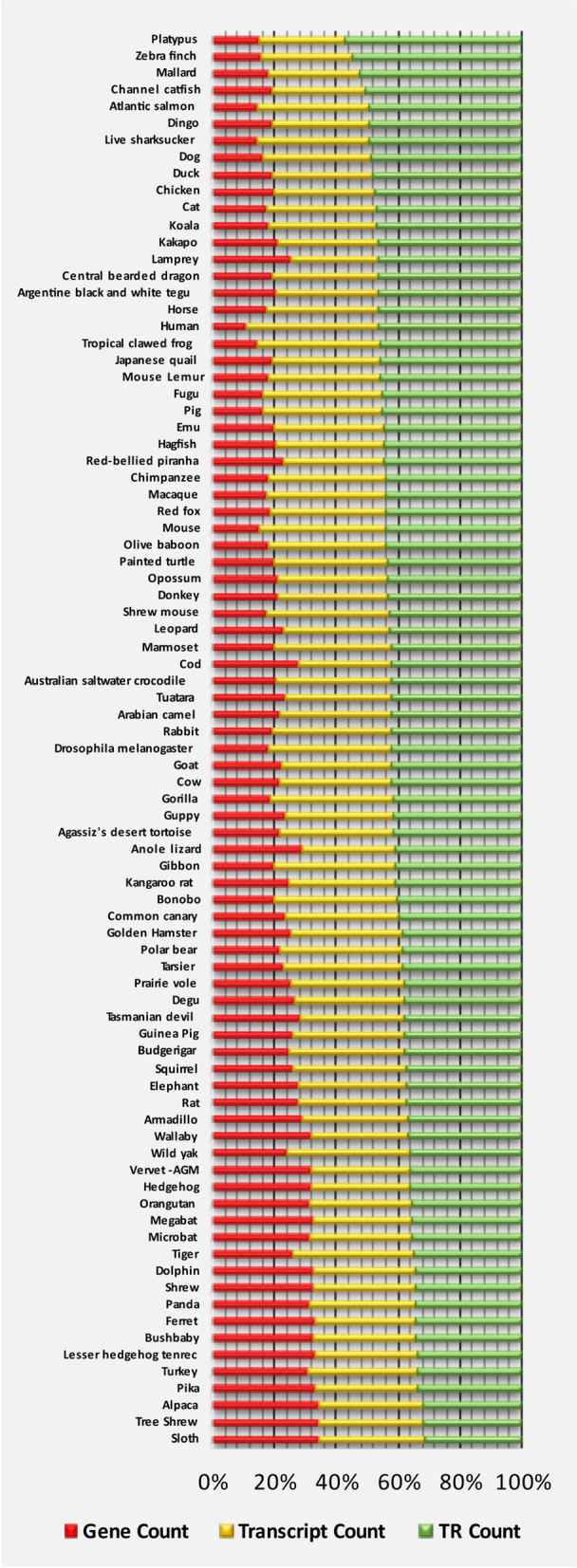
Ratios of genes, transcripts, and TR counts for each species. The horizontal axis shows the percentage of each entity, and the vertical axis shows each species. Species can be cross-referenced in Additional Table 1

A total of 1,611,368 protein-coding genes, 2,730,515 transcripts and 3,283,771 TRs were investigated across the 84 selected species, of which 22,791 genes, 93,706 transcripts, and 99,818 TRs belonged to the human species (Additional Table 1). On average, there were 1.64 transcripts and 1.97 TRs per gene, and 1.19 TRs, per transcript, per species (Fig. 1). The highest ratios of transcripts and TRs per gene (4.11 and 4.38, respectively) belonged to human. Human ranked 59th among 84 species in respect of the TR/transcript ratio (Fig. 2) (Additional Table 1).

Across the 93,706 identified protein-coding transcripts in the human genome, there were 50,169 transcripts, in which TISs were flanked by at least one TR (53.54% of protein-coding transcripts). At a similarly high rate, from the 22,791 identified protein-coding genes in the human genome, 15,256 genes had at least one transcript, in which TISs were flanked by a TR (66.94% of human protein-coding genes). 2850 different types of TRs were identified in the human genome, of which 1504 types (52.77%) were human-specific; across TR categories 1–4, we detected 660, 101, 339 and 404 types of human-specific TRs, respectively, the top most abundant of which are represented in Table 1.

**Table 1:** The top most abundant human-specific TRs flanking TISs. It should be noted that human-specificity applied in the context of the relevant TISs

	Tandem Repeat	Core Length
**Category 1**	(CT)3	2
	(TC)3	2
	(GC)3	2
	(T)6	1
	(CG)3	2
	(GGC)3	3
	(CTG)3	3
	(CGCC)3	4
	(GGGGC)3	5
	(TGTTTT)3	6
	(CGCGCC)3	6
**Category 2**	(GGGGCGC)3	7
	(CCCGCCG)4	7
	(GCTGCGGG)3	8
	(AGGGGCGGG)4	9
	(CCTCCCG)4	7
	(CCGGGGG)3	7
	(TTTTTTG)3	7
	(AGCCCAGC)3	8
	(CCCCCGC)3	7
	(ACCCCTCC)3	8
	(AGCCCACGG)3	9
**Category 3**	(GTGTGTGTTT)2	10
	(ATTTTAAAATT)2	11
	(AAAATAAATAA)2	11
	(TGGCGGCGGCGG)2	12
	(CCCAGCCCCA)2	10
	(CCCCGCCCGCG)2	11
	(CGGGAGTGAGAG)2	12
	(AAGTGGGAAACTGG)2	14
	(TTCATAGATGTTC)2	13
	(ATAGATGTTC)2	10
	(CCCCGCCCCT)2	10
**Category 4**	(CCCCGAGGTCTCCGCG)2	16
	(CCGGCGTGTACCGAGAGACTGGCGT)2	25
	(ACCTGGAGGGCTGGGG)2	16
	(CCCTGCCCTGTCCTGTCCTGCCCTG)2	25
	(ACCCATCCCCACCTCCCT)3	18
	(CCCTGCCCTGTCCTGTCCTG)2	20
	(ACCCATCCCCACCTCCCT)3	18
	(CCCCACCTCCCTACCCAT)4	18
	(ACAGCGAGGTCGGCAGCGGCAGCGAGGTCGGCAGCGGC)2	38
	(TGAGTCGCAGGCCGAGGAGACAGTGAGTGCGCGCCC)2	36
	(ACTCTCTCTCTTTCTCGGGCTGCAGGTGCACCAGGCCGTCC)2	41

### TRs differentially co-occur with TISs

**Fig. 3:**
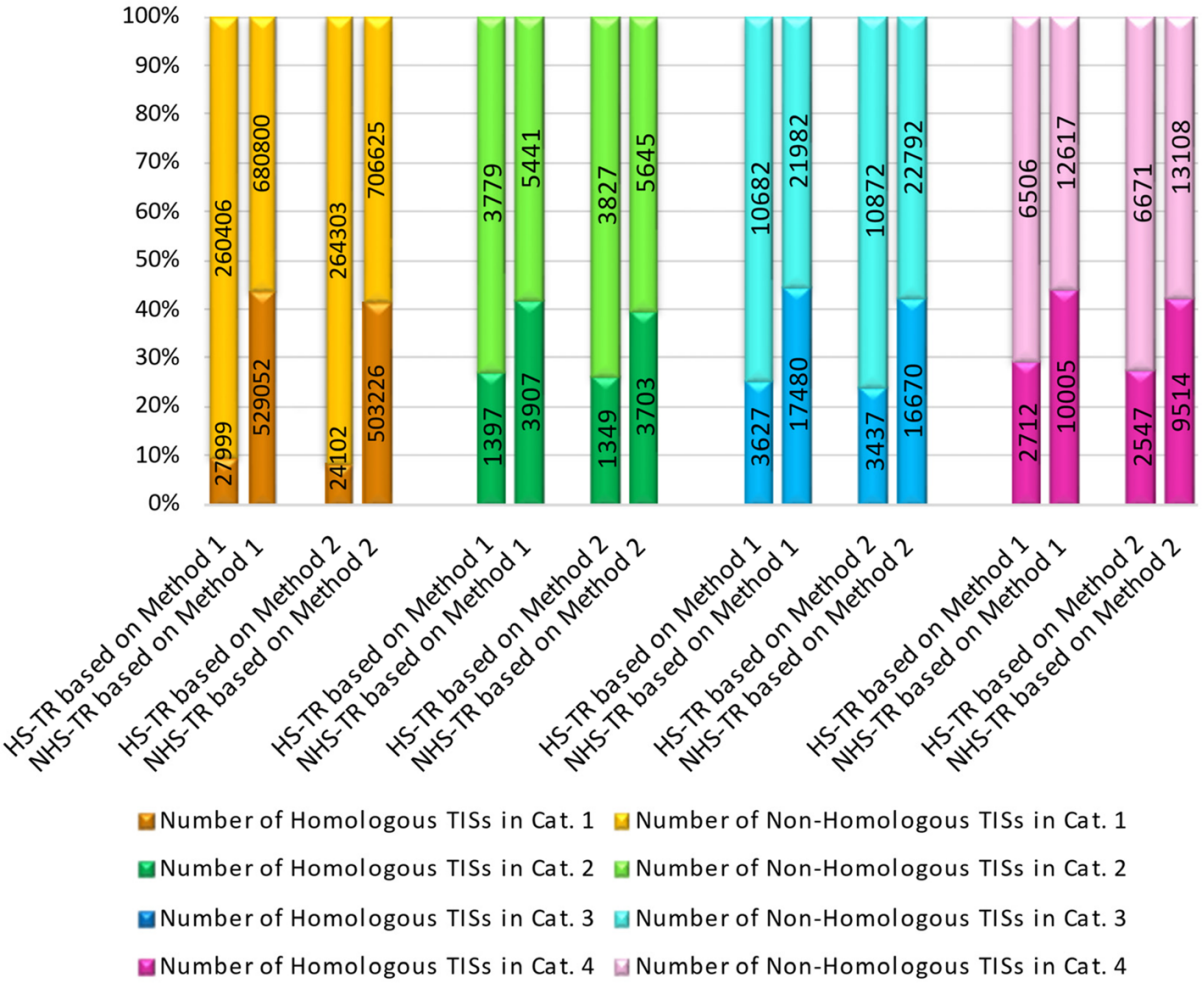
Average of 10 experiments to examine co-occurrence patterns between TRs and TISs in each of the four TR categories. Each histogram shows the number of homologous vs. non-homologous TISs, based on two different weighing methods (vectors). HS-TR = human-specific tandem repeat, NHS-TR = non-human-specific tandem repeat, TIS = translation initiation site

**Fig. 4:**
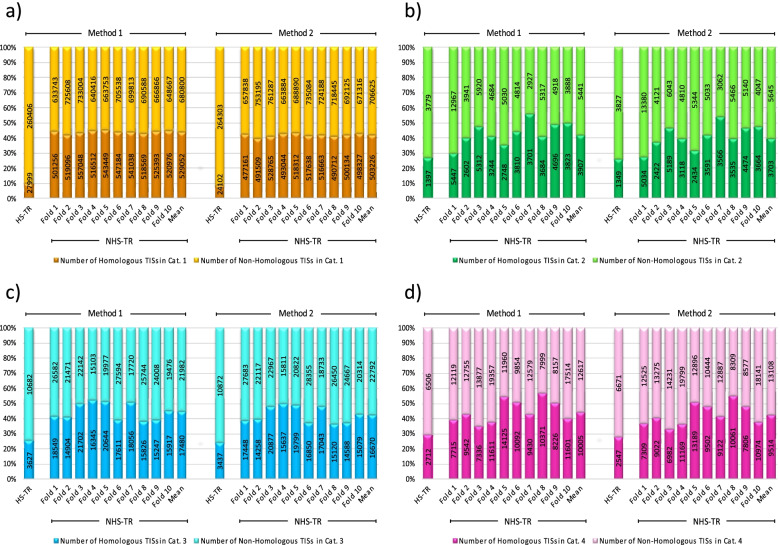
10-fold cross-validation of co-occurrence patterns between TRs and TISs in TR Categories 1–4. Each histogram shows the number of homologous vs. non-homologous TISs, based on two different weighing methods (vectors), as follows: category 1 (a), category 2 (b), category 3 (c), and category 4 (d) (Please see text for the description of TR categories 1 to 4). HS-TR = human-specific tandem repeat, NHS-TR = non-human-specific tandem repeat, TIS = translation initiation site

We employed two weighing settings (vectors) for designating homologous vs. non-homologous TISs in human vs. other species. One of those settings was the same as in our previous approach (vector *W*_1_) [33]. In both settings, there was significant co-occurrence of human-specific TRs with non-homologous human TISs, and non-human-specific TRs with homologous human TISs (Fisher’s exact *p* < 0.01) (Fig. 3). The results were replicated in 10-fold cross-validation (Fig. 4) (Additional Table 2).

### Biological and evolutionary implications

In 15,256 human genes, at least one TIS was flanked by a TR, of which in 2991 genes those TRs were human-specific (Additional Tables 3 & 4). A sample of those genes is listed in Table 2, text mining [34] of a number of which yielded predominant expression and functions in the human brain and/or skeletal muscle, such as *CACNA1A*, *EIF5AL1*, *FOXK1*, *GABRB2, MYH2*, *SLC6A8*, and *TTN*. These are examples of expression enrichment in tissues that are frequently subject to human-specific evolutionary processes. However, the nervous system and skeletal muscle may not be the only tissues, gene functions in which are associated with human-specific characteristics.

**Table 2:** Example of human genes (represented by *gene symbol*), which contain human-specific TRs

No.	Gene Symbol	No.	Gene Symbol	No.	Gene Symbol	No.	Gene Symbol
1	*ACSL6*	28	*DMPK*	55	*KRT23*	82	*PHF8*
2	*ADAM22*	29	*DOK6*	56	*KRT73*	83	*PLEC*
3	*ADSSL1*	30	*EFHC1*	57	*KRT8*	84	*PPP1CC*
4	*AKAP7*	31	*EIF3K*	58	*L3MBTL1*	85	*PPP1R14A*
5	*ARHGAP42*	32	*EIF5AL1*	59	*LCAT*	86	*PRIMA1*
6	*ASIC1*	33	*ELMO1*	60	*LMNA*	87	*PTBP1*
7	*ASRGL1*	34	*ENSG00000258947*	61	*MBNL1*	88	*REG1B*
8	*ATXN10*	35	*EPB41L4B*	62	*MPRIP*	89	*RYR1*
9	*C11orf63*	36	*EXTL3*	63	*MTDH*	90	*RYR3*
10	*C19orf12*	37	*FAM101B*	64	*MYH2*	91	*SCIN*
11	*CACNA1A*	38	*FMNL3*	65	*NEK3*	92	*SERHL2*
12	*CACNA1F*	39	*FOXK1*	66	*NOL3*	93	*SERPINB6*
13	*CACNA1G*	40	*FOXP1*	67	*OBSCN*	94	*SIPA1L3*
14	*CAPNS2*	41	*GABRB2*	68	*OLIG1*	95	*SLC25A27*
15	*CDK16*	42	*GDF11*	69	*PAMR1*	96	*SLC4A1*
16	*CELF4*	43	*GSK3A*	70	*PANK2*	97	*SLC6A8*
17	*CELF6*	44	*GSTM2*	71	*PCDH7*	98	*SLIT2*
18	*CEP55*	45	*HCN2*	72	*PCDHA10*	99	*SPEG*
19	*CERCAM*	46	*HDAC4*	73	*PCDHA12*	100	*SYN1*
20	*CKB*	47	*HDAC8*	74	*PCDHA13*	101	*SYNGAP1*
21	*CLIP2*	48	*HRC*	75	*PCDHA7*	102	*TCF3*
22	*COL3A1*	49	*INPP5K*	76	*PCDHB14*	103	*TMEM132A*
23	*COPRS*	50	*ITSN1*	77	*PCDHB5*	104	*TMEM59L*
24	*CRIPT*	51	*KCNA2*	78	*PCDHB6*	105	*TRNP1*
25	*CROCC*	52	*KCNC1*	79	*PCDHB9*	106	*TTN*
26	*DAO*	53	*KIAA1191*	80	*PCDHGC4*	107	*ZFHX3*
27	*DCTN2*	54	*KRT10*	81	*PDLIM4*		

**Fig. 5:**
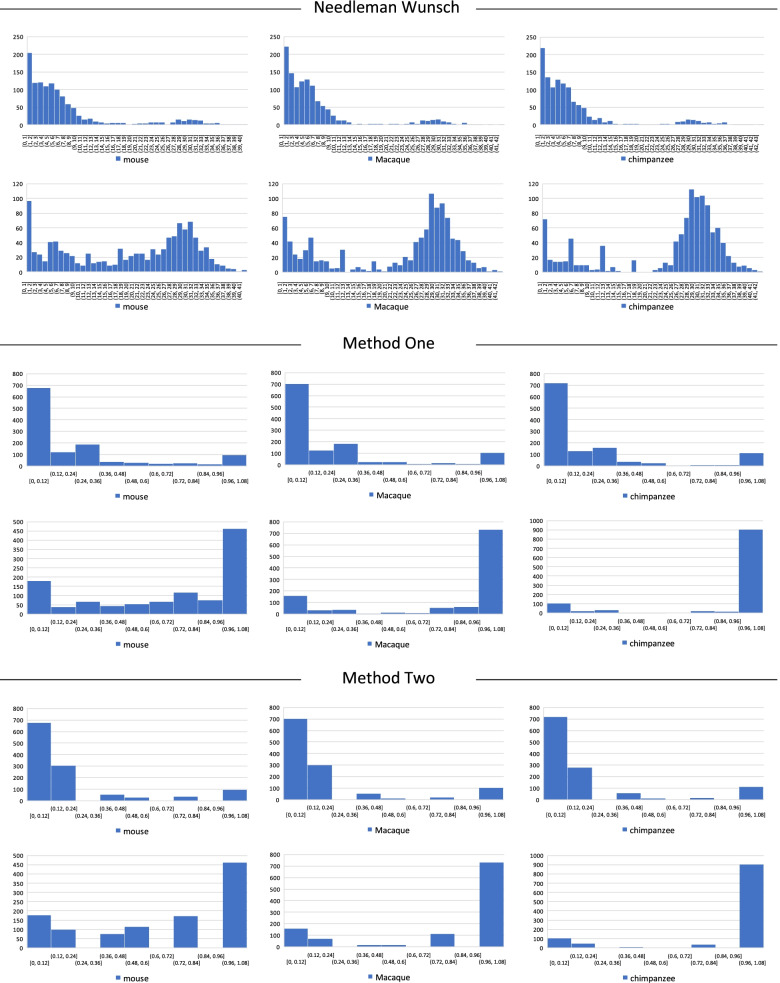
Protein homology check of TISs flanked by human-specific and non-specific TRs. Every chart shows the distribution of similarity abundance between human proteins and three species, mouse, macaque, and chimpanzee, in the same gene. For each panel, the first row shows the distribution that was constructed by BLASTing human proteins, TISs of which were flanked by human-specific TRs. Similarly, the second row of each panel shows the distribution that was constructed by BLASTing human proteins, TISs of which were flanked by non-human-specific TRs. The Needleman Wunsch algorithm (upper panel) was used as a complementary measure to our two weighing methods (methods 1 and 2). In each method, we detected a significant difference in the distribution. TIS = translation initiation site, TR = tandem repeat

We employed the Needleman Wunsch algorithm [35] to further examine the relevance of our findings. To that end, comparison of proteins between human and three other species, consisting of chimpanzee, macaque, and mouse (RESTful API at: https://www.ebi.ac.uk/Tools/psa/emboss_needle [36]), revealed significantly lower homology for the human proteins, in which TISs were flanked by human-specific TRs (Fig. 5).

Our findings provide prime evidence of a link between TRs of all core lengths and repeats, and TIS selection, mechanisms of which are virtually unknown currently. Our approach was based on homology search, which reliably identifies” homologous” TISs by detecting excess similarity [37]. By searching identical gene names across the selected species, our approach encompassed orthologous and paralogous genes.

While the scope of our previous publication [33] was limited to the STRs, in the current study, we investigated TRs of all core lengths (ranging from 1 to 60 nucleotides) and repeats. Another advantage was employment of an improved method for retrieving the upstream flanking sequences. Moreover, whereas BLAST of CDS and cDNA sequences were used to extract the TISs and upstream flanking sequences in the previous study, here we used script programming on the Biomart web application, which is more reliable and accurate. In this method, we specified the gene name, transcript, and length of the upstream flanking sequence for the Biomart web application [38], by using an automated script. In comparison with our previously implemented methods, the result of the automated script is more accurate and comprehensive. An additional weighing method was also implemented in the current study to further examine the relevance of our homology assignment approach.

It is possible that asymmetric and stem-loop structures, which are inherent properties of repeat sequences result in genetic marks that enhance TIS selection. Asymmetric structures have recently been reported to be linked to various biological functions, such as replication and initiation of transcription start sites [39]. Recent studies implicate that the local folding and co-folding energy of the ribosomal RNA (rRNA) and the mRNA correlates with codon usage estimators of expression levels in model organisms such as chloroplast [40]. It may be speculated that RNA structures formed as a result of folding in the TR regions function as marks for TISs.

Among a number of options for future studies, genome editing strategies such as CRISPR/Cas9 [41] in combination with in vitro translation engineering, using cell-free protein synthesis (also known as *in vitro* protein synthesis or CFPS) and/or PURE system (i.e. protein synthesis using purified recombinant elements) [42, 43] may be useful to investigate the impact of TRs on TIS selection and protein synthesis.

## Conclusion

We conclude that TRs ubiquitously flank TIS sequences and contribute to TIS selection at the trans-species level. Future functional analyses, such as a combination of genome editing strategies and in vitro protein synthesis are warranted to investigate the impact of TRs on TIS selection.

## Materials and methods

### Data collection

**Fig. 6:**
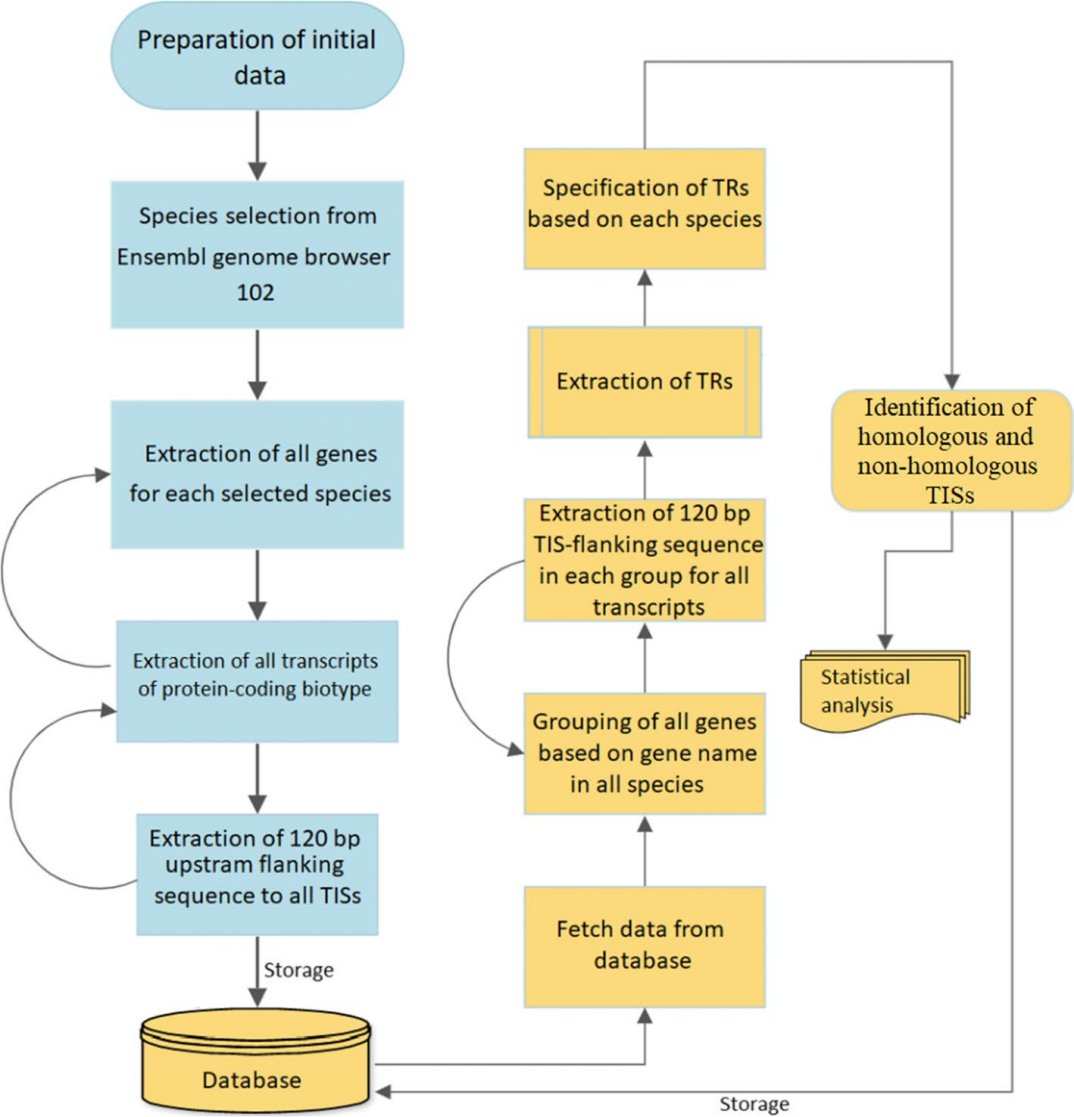
Scheme representing the steps taken for data collection and analysis

All sequences, species, and gene datasets collected in this study were based on Ensembl 102 (http://nov2020.archive.ensembl.org/index.html), scheme of which is depicted in Fig. 6 .

84 species were selected, which encompassed orders of vertebrates and one non-vertebrate species (*D. melanogaster*) (Fig. 2). Throughout the study, all species were compared with the human sequence, as reference. The list of species was extracted via RESTful API, in Java language. In parallel, a list of available gene datasets of the selected species was collected by using the “biomaRt” package [44, 45] in R language. In the next step, in each selected species, all protein-coding transcripts of protein-coding genes were extracted. To that end, identical gene names were used across the selected species to group orthologous/paralogous genes in those species.

Subsequently, the 120 bp upstream flanking sequence of all annotated protein coding TISs were retrieved and analyzed. All steps of data collection were performed by querying on the Biomart Ensembl tool via RESTful API, which was implemented in the Java language, except fetching the primary list of available species and gene datasets. For each species, its name, common name and display name were retrieved. For each gene in each species, its gene name, Ensembl ID and the annotated transcript IDs were retrieved, and finally, for each transcript, the coding sequence, the TIS, the upstream flanking sequence of the TIS, and the protein sequence were retrieved.

All collected data was stored in a MySQL database which is accessible at https://figshare.com/search?q=10.6084%2Fm9.figshare.15405267 .

A candidate sequence was considered a TR if it complied with the following four rules: (1) for mononucleotide cores, the number of repeats should be ≥6. (2) for 2–9 bp cores, the number of repeats should be ≥3. (3) for other core lengths, the number of repeats should be ≥2. (4) TRs of the same core sequence should not overlap if they were in the same upstream flanking sequence.

We categorized the TRs based on the core lengths as follows: Category 1: 1–6 bp, Category 2: 7–9 bp, Category 3: 10–15 bp, and Category 4: ≥16 bp. This was an arbitrary classification to allow for possible differential effect of various core length ranges in evolutionary and biological terms.

### Retrieval of data across species

Using the enhanced query (Additional Table 5) form on the Biomart Ensembl tool along with the RESTful API tools, a Java package was developed to retrieve, store, and analyze the data and information. The source codes and the Java package are available at: https://github.com/Yasilis/STRsMiner-JavaPackage_PaperSubmission/tree/develop .

### Identification of human-specific TRs

The 120 bp upstream flanking sequence of TISs of all annotated protein-coding transcripts of protein-coding genes were screened in 84 species for the presence of TRs in four categories based on the TR core length. The data obtained on the human TRs was compared to those of other species, and the TRs which were specific to human were identified.

To identify human-specific TRs, in the first step, the selected genes of all species were grouped based on gene name. Therefore, all homologous genes, consisting of orthologous and paralogous genes, were placed in one group. In each group, all the TRs located in the upstream flanking sequence of every transcript were extracted. In the next step, the extracted TRs were grouped and specified according to the species. All the TRs that were detected in more than one species were removed. The remaining TRs belonged to only one species and were specific to that species. Subsequently, we identified the human-specific TRs for a specific gene name by selecting the human species. This process was repeated for each group of genes and the results were aggregated together to identify all the TRs which were specific and non-specific in reference to human.

### Evaluation of TIS homology

Identifying the degree of homology between two transcripts requires assigning a weight value to each position of the sequence. Weighted homology scoring was performed in two different weight settings, as weighing vectors *W*_1_ (originally used by our group for studying a link between STRs and TIS selection) [33] and *W*_2_, which can be distinguished by *k* = {1, 2}. These two weighing vectors are defined as follow (Eq. 1, 2):

1 $${W}_1=\left\{0,25,25,25,12.5,12.5\Big\}\right.$$

2 $${W}_2=\left\{0,20,20,20,20,20\Big\}\right.$$

If M is the first methionine amino acid of the two peptide sequences (position of 0 in the two weighing vectors), for all next five successive positions represented by *i* in the formula (Eq. 9), we defined five weight coefficients *w*_*k*, 1_ to *w*_*k*, 5_, observed in the *W*_*k*_ vector.

Homology of the first five amino acids (excluding the initial methionine), and, therefore the TIS, was inferred based on the value of pair-wise similarity scoring between human, as reference, and other species. A similarity of ≥50% was considered “homology”. This threshold was achieved following BLASTing three thousand random pair-wise similarity checks of the initial five amino acids of randomly selected proteins as previously described [33].

### Scoring human-specific and non-specific TR co-occurrences with homologous and non-homologous TISs

In both weighing methods, the initial five amino acid sequence (excluding the initial methionine) of the human TISs that were flanked by human-specific and non-specific TRs were BLASTed against all the initial five amino acids (excluding the initial methionine) of the orthologous/paralogous genes in the remaining 83 species. The above was aimed at comparing the number of events in which human-specific and non-specific TRs co-occurred with homologous and non-homologous (TISs) in reference to human. For computing the number of homologous and non-homologous TISs, we needed to consider a number of assumptions. We defined G as the set of all human protein coding genes. Therefore, *g* denoted a gene that belonged to the G set (Eq. 3).

3 $$G=\left\{g|g\ is\ a\ human\ protein\ coding\ gene\right\}$$

We also defined *T*_*H*_(*g*) and ${T}_{\overline{H}}(g)$ as the set of all annotated transcripts in a gene *g*, which belonged to human and other species, respectively (Eqs. 4 and 5).

4 $${T}_H(g)=\left\{t\ |\begin{array}{c}\ t\ \mathrm{was}\ \mathrm{a}\ \mathrm{human}\ \mathrm{protein}\ \mathrm{coding}\ \mathrm{transcript}\ \mathrm{which}\ \\ {}\mathrm{belonged}\ \mathrm{to}\ \mathrm{the}\ \mathrm{gene},g\end{array}\right\}$$

5 $${T}_{\overline{H}}(g)=\left\{t\ |\begin{array}{c}\ t\ \mathrm{was}\ \mathrm{a}\ \mathrm{protein}\ \mathrm{coding}\ \mathrm{transcript}\ \mathrm{which}\ \mathrm{belonged}\ \\ {}\ \mathrm{to}\ \mathrm{the}\ \mathrm{gene},g\ \mathrm{but},\mathrm{did}\ \mathrm{not}\ \mathrm{exist}\ \mathrm{in}\ \mathrm{human}\end{array}\right\}$$

Moreover, *T*^∗^ denoted all filtered transcripts of *T* which had at least one human-.

specific TR at the 120 bp interval upstream of the TIS, while, *T*^+^ denoted all filtered transcripts of *T*, which had at least one TR at the 120 bp interval upstream of the TIS.

The following formula was developed to measure the degree of similarity of two peptides in the two weighing settings (Eq. 6).

6 $${H}_k=\sum_{g\epsilon G\ }\sum_{t_a\epsilon {T}_H^{\ast }(g)\ }\sum_{t_b\epsilon {T}_{\overline{H}}^{+}(g)\ }{\Theta}_k\left({t}_a,{t}_b\right)$$

In this formula, Θ is a binary function that decides whether the transcripts are homologous or not, and *k* = {1, 2} refer to each weight setting. If *S* function measures the similarity score, Θ can be defined as follow (Eq. 7):

7 $${\Theta}_k\left({t}_a,{t}_b\right)=\left\{\ \begin{array}{c}1, if\ {S}_k\left({t}_a,{t}_b\right)\ge 50\\ {}0,o.w.\end{array}\right.$$

For calculating the similarity score, we used another binary function. We defined Φ as follows: (Eq. 8):

8 $$\Phi \left(x,y\right)=\left\{\begin{array}{c}1, if\ x=y\\ {}0,o.w.\end{array}\right.$$

This function takes two amino acids as argument and returns 1 as output if they are the same, and zero if they are not the same. Therefore, *S*(*t*_*a*_, *t*_*b*_) is defined by the following formula (Eq. 9):

9 $${S}_k\left({t}_a,{t}_b\right)=\sum_{i=2}^6{w}_{k,i}\Phi \left({P}_i\left({t}_a\right),{P}_i\left({t}_b\right)\ \right)$$

In this function, the *i*^*th*^ amino acid in the sequence of the transcript *t*, is denoted by *P*_*i*_(*t*).

We replicated the comparisons in 10-fold cross-validation. In each-fold, genes with human non-specific TRs were randomly selected according to the number of genes in the group with human-specific TRs. This process was repeated for the two methods (two different weight vectors) and for each of the four categories of TRs. For each category and weighing method, the mean of the result of each round was calculated as a final result. Finally, the Fisher’s exact test was run for each-fold (Additional Table 2).

## Supplementary Information

**Additional file 1 Additional Table 1.** The number of genes, transcripts and extracted TRs for each species. The rows of the table are sorted from large to small, based on the ratio of the number of TRs to the number of genes and transcripts in each species.

**Additional file 2 Additional Table 2.** The number of events/co-occurrences of homologous and non-homologous TISs (in human as reference) with the two groups of human-specific and non-specific TRs and their *p*-values, calculated by Fisher’s exact test in each method across TR categories 1, 2, 3 and 4.

**Additional file 3 Additional Table 3.** The list of all human genes and their Ensembl gene ID, which contained human-specific TRs in their TIS-flanking sequence for TR categories 1, 2, 3, and 4.

**Additional file 4 Additional Table 4.** The list of all human specific TRs and their abundance.

**Additional file 5 Additional Table 5.** The list of queries that were used to communicate with the Ensembl data repositories.

## Data Availability

The datasets generated and analyzed during this study are available in the “figshare” repository, with the identifier “10.6084/m9.figshare.15405267”. Also, other source code and software available in the GitHub repository. (https://github.com/Yasilis/STRsMiner-JavaPackage_PaperSubmission/tree/develop)

## Abbreviations

TIS: Translation initiation site
TR: Tandem repeat
STR: Short tandem repeat
ORF: Open reading frame
UTR: untranslated region
HS-TR: Human-specific TR
NHS-TR: Non-human-specific TR
