# Supplementary material for: Tandem repeats ubiquitously flank and contribute to translation initiation sites
Source: BMC Genom Data. 2022 Jul 27;23:59. doi: 10.1186/s12863-022-01075-5 (PMC9331589; doi:10.1186/s12863-022-01075-5)
Supplement: Supplementary file 2 — Additional file 2 Additional Table 2. The number of events/co-occurrences of homologous and non-homologous TISs (in human as reference) with the two groups of human-specific and non-specific TRs and their p-values, calculated by Fisher’s exact test in each method across TR categories 1, 2, 3 and 4. [file 12863_2022_1075_MOESM2_ESM.docx]

|  | Category 1 | | | | | |
| --- | --- | --- | --- | --- | --- | --- |
|  |  | HS-TRs | | NHS-TRs | |  |
|  | Fold Number | Number of Homologous events | Number of non-Homologous events | Number of Homologous events | Number of non-Homologous events | p-value* |
| Method 1 | 1 | 27999 | 260406 | 501256 | 633743 | 0 |
|  | 2 |  |  | 519096 | 725608 | 0 |
|  | 3 |  |  | 557048 | 733004 | 0 |
|  | 4 |  |  | 516512 | 640416 | 0 |
|  | 5 |  |  | 543449 | 663753 | 0 |
|  | 6 |  |  | 547184 | 705538 | 0 |
|  | 7 |  |  | 541038 | 699813 | 0 |
|  | 8 |  |  | 518569 | 690588 | 0 |
|  | 9 |  |  | 525393 | 666866 | 0 |
|  | 10 |  |  | 520976 | 648667 | 0 |
|  | Mean |  |  | 529052 | 680800 | 0 |
| Method 2 | 1 | 24102 | 264303 | 477161 | 657838 | 0 |
|  | 2 |  |  | 491509 | 753195 | 0 |
|  | 3 |  |  | 528765 | 761287 | 0 |
|  | 4 |  |  | 493044 | 663884 | 0 |
|  | 5 |  |  | 518312 | 688890 | 0 |
|  | 6 |  |  | 517638 | 735084 | 0 |
|  | 7 |  |  | 516663 | 724188 | 0 |
|  | 8 |  |  | 490712 | 718445 | 0 |
|  | 9 |  |  | 500134 | 692125 | 0 |
|  | 10 |  |  | 498327 | 671316 | 0 |
|  | Mean |  |  | 503226 | 706625 | 0 |

|  | Category 2 | | | | | |
| --- | --- | --- | --- | --- | --- | --- |
|  |  | HST-TRs | | NHS-TRs | |  |
|  | Fold Number | Number of Homologous events | Number of non-Homologous events | Number of Homologous events | Number of non-Homologous events | p-value |
| Method 1 | 1 | 1397 | 3779 | 5447 | 12967 | 0.0001424671 |
|  | 2 |  |  | 2602 | 3941 | 2.796959e-48 |
|  | 3 |  |  | 5312 | 5920 | 9.719377e-138 |
|  | 4 |  |  | 3244 | 4684 | 6.13069e-61 |
|  | 5 |  |  | 2748 | 5030 | 7.160126e-24 |
|  | 6 |  |  | 3810 | 4814 | 1.093386e-92 |
|  | 7 |  |  | 3701 | 2927 | 1.477926e-221 |
|  | 8 |  |  | 3684 | 5317 | 7.990065e-64 |
|  | 9 |  |  | 4696 | 4918 | 1.305653e-150 |
|  | 10 |  |  | 3823 | 3888 | 3.567747e-148 |
|  | Mean |  |  | 3907 | 5441 | 2.995902e-72 |
| Method 2 | 1 | 1349 | 3827 | 5034 | 13380 | 0.03511037 |
|  | 2 |  |  | 2422 | 4121 | 5.366279e-37 |
|  | 3 |  |  | 5189 | 6043 | 5.826668e-137 |
|  | 4 |  |  | 3118 | 4810 | 1.815834e-56 |
|  | 5 |  |  | 2434 | 5344 | 6.859337e-11 |
|  | 6 |  |  | 3591 | 5033 | 3.602719e-78 |
|  | 7 |  |  | 3566 | 3062 | 3.864741e-207 |
|  | 8 |  |  | 3535 | 5466 | 1.356952e-58 |
|  | 9 |  |  | 4474 | 5140 | 1.39143e-134 |
|  | 10 |  |  | 3664 | 4047 | 7.598915e-136 |
|  | Mean |  |  | 3703 | 5645 | 3.640182e-62 |

|  | Category 3 | | | | | |
| --- | --- | --- | --- | --- | --- | --- |
|  |  | HS-TRs | | NHS-TRs | |  |
|  | fold number | Number of Homologous events | Number of non-Homologous events | Number of Homologous events | Number of non-Homologous events | p-value |
| Method 1 | 1 | 3627 | 10682 | 18549 | 26582 | 3.388754e-263 |
|  | 2 |  |  | 14904 | 21471 | 7.257548e-246 |
|  | 3 |  |  | 21702 | 22142 | 0 |
|  | 4 |  |  | 16345 | 15103 | 0 |
|  | 5 |  |  | 20644 | 19977 | 0 |
|  | 6 |  |  | 17611 | 27594 | 2.228011e-200 |
|  | 7 |  |  | 18056 | 17720 | 0 |
|  | 8 |  |  | 15826 | 25744 | 2.988572e-173 |
|  | 9 |  |  | 15247 | 24008 | 1.205477e-190 |
|  | 10 |  |  | 15917 | 19476 | 0 |
|  | Mean |  |  | 17480 | 21982 | 0 |
| Method 2 | 1 | 3437 | 10872 | 17448 | 27683 | 2.747628e-234 |
|  | 2 |  |  | 14258 | 22117 | 1.277096e-237 |
|  | 3 |  |  | 20877 | 22967 | 0 |
|  | 4 |  |  | 15637 | 15811 | 0 |
|  | 5 |  |  | 19799 | 20822 | 0 |
|  | 6 |  |  | 16850 | 28355 | 7.418423e-195 |
|  | 7 |  |  | 17043 | 18733 | 0 |
|  | 8 |  |  | 15120 | 26450 | 1.188811e-167 |
|  | 9 |  |  | 14588 | 24667 | 1.755099e-185 |
|  | 10 |  |  | 15079 | 20314 | 0 |
|  | Mean |  |  | 16670 | 22792 | 0 |

|  | Category 4 | | | | | |
| --- | --- | --- | --- | --- | --- | --- |
|  |  | HS-TRs | | NHS-TRs | |  |
|  | fold number | Number of Homologous events | Number of non-Homologous events | Number of Homologous events | Number of non-Homologous events | p-value |
| Method 1 | 1 | 2712 | 6506 | 7715 | 12119 | 1.662609e-56 |
|  | 2 |  |  | 9542 | 12755 | 1.820374e-111 |
|  | 3 |  |  | 7336 | 13877 | 4.990456e-19 |
|  | 4 |  |  | 11611 | 19357 | 5.720837e-47 |
|  | 5 |  |  | 14125 | 11960 | 0 |
|  | 6 |  |  | 10092 | 9854 | 4.570111e-258 |
|  | 7 |  |  | 9430 | 12579 | 7.26912e-112 |
|  | 8 |  |  | 10371 | 7999 | 0 |
|  | 9 |  |  | 8226 | 8157 | 9.033439e-234 |
|  | 10 |  |  | 11601 | 17514 | 1.261387e-74 |
|  | Mean |  |  | 10005 | 12617 | 1.343825e-135 |
| Method 2 | 1 | 2547 | 6671 | 7309 | 12525 | 4.567926e-55 |
|  | 2 |  |  | 9022 | 13275 | 2.119927e-105 |
|  | 3 |  |  | 6982 | 14231 | 2.187843e-20 |
|  | 4 |  |  | 11169 | 19799 | 2.772763e-52 |
|  | 5 |  |  | 13189 | 12896 | 0 |
|  | 6 |  |  | 9502 | 10444 | 2.96591e-235 |
|  | 7 |  |  | 9122 | 12887 | 1.331695e-120 |
|  | 8 |  |  | 10061 | 8309 | 0 |
|  | 9 |  |  | 7806 | 8577 | 4.99406e-221 |
|  | 10 |  |  | 10974 | 18141 | 1.531816e-71 |
|  | Mean |  |  | 9514 | 13108 | 9.104462e-132 |

*p-values are represented exactly based on package R.
