# Supplementary material for: Tandem repeats ubiquitously flank and contribute to translation initiation sites
Source: BMC Genom Data. 2022 Jul 27;23:59. doi: 10.1186/s12863-022-01075-5 (PMC9331589; doi:10.1186/s12863-022-01075-5)
Supplement: Supplementary file 5 — Additional file 5 Additional Table 5. The list of queries that were used to communicate with the Ensembl data repositories. [file 12863_2022_1075_MOESM5_ESM.docx]

|  | The list of queries for fetching data | |
| --- | --- | --- |
| 1 | Getting the list of all species' names in Ensembl database | http://rest.ensembl.org/info/species?content-type=application/json |
| 2 | Getting the list of all genes from specific gene datasets (e.g. hsapiens_gene_ensembl) | http://www.ensembl.org/biomart/martservice?query=%3C?xml%20version=%221.0%22%20encoding=%22UTF-8%22?%3E%3C!DOCTYPE%20Query%3E%3CQuery%20virtualSchemaName%20=%20%22default%22%20formatter%20=%20%22TSV%22%20header%20=%20%220%22%20uniqueRows%20=%20%220%22%20count%20=%20%22%22%20datasetConfigVersion%20=%20%220.6%22%20%3E%3CDataset%20name%20=%20%22 + ***<the name of gene dataset>*** + %22%20interface%20=%20%22default%22%20%3E%3CFilter%20name%20=%20%22biotype%22%20value%20=%20%22protein_coding%22/%3E%3CAttribute%20name%20=%20%22ensembl_gene_id%22%20/%3E%3CAttribute%20name%20=%20%22gene_biotype%22%20/%3E%3CAttribute%20name%20=%20%22external_gene_name%22%20/%3E%3C/Dataset%3E%3C/Query%3E |
| 3 | Getting the list of the 120 bp upstream flanking sequence of specific genes Ensemble ID in its gene dataset (e.g. hsapiens_gene_ensembl, ENSG00000180016) | http://www.ensembl.org/biomart/martservice?query="%3C?xml%20version=%221.0%22%20encoding=%22UTF-8%22?%3E%3C!DOCTYPE%20Query%3E%3CQuery%20%20virtualSchemaName%20=%20%22default%22%20formatter%20=%20%22FASTA%22%20header%20=%20%220%22%20uniqueRows%20=%20%220%22%20count%20=%20%22%22%20datasetConfigVersion%20=%20%220.6%22%20%3E%3CDataset%20name%20=%20%22 + ***<the name of gene dataset>*** + %22%20interface%20=%20%22default%22%20%3E%3CFilter%20name%20=%20%22upstream_flank%22%20value%20=%20%22120%22/%3E%3CFilter%20name%20=%20%22transcript_biotype%22%20value%20=%20%22protein_coding%22/%3E%3CFilter%20name%20=%20%22biotype%22%20value%20=%20%22protein_coding%22/%3E%3CFilter%20name%20=%20%22ensembl_gene_id%22%20value%20=%20%22 + ***<gene Ensembl ID>*** + %22/%3E%3CAttribute%20name%20=%20%22ensembl_gene_id%22%20/%3E%3CAttribute%20name%20=%20%22ensembl_transcript_id%22%20/%3E%3CAttribute%20name%20=%20%22coding_transcript_flank%22%20/%3E%3C/Dataset%3E%3C/Query%3E |
| 4 | Getting the peptide's sequence for specific transcript Ensemble ID in its gene dataset (e.g. hsapiens_gene_ensembl, ENST00000000233) | http://www.ensembl.org/biomart/martservice?query=%3C?xml%20version=%221.0%22%20encoding=%22UTF-8%22?%3E%3C!DOCTYPE%20Query%3E%3CQuery%20%20virtualSchemaName%20=%20%22default%22%20formatter%20=%20%22FASTA%22%20header%20=%20%220%22%20uniqueRows%20=%20%220%22%20count%20=%20%22%22%20datasetConfigVersion%20=%20%220.6%22%20%3E%3CDataset%20name%20=%20%22 + ***<the name of gene dataset>*** + %22%20interface%20=%20%22default%22%20%3E%3CFilter%20name%20=%20%22transcript_biotype%22%20value%20=%20%22protein_coding%22/%3E%3CFilter%20name%20=%20%22ensembl_transcript_id%22%20value%20=%20%22 + ***<transcript Ensembl ID>*** + %22/%3E%3CAttribute%20name%20=%20%22peptide%22%20/%3E%3CAttribute%20name%20=%20%22ensembl_gene_id%22%20/%3E%3CAttribute%20name%20=%20%22ensembl_transcript_id%22%20/%3E%3C/Dataset%3E%3C/Query%3E |
